# NMR and IR Investigations of Strong Intramolecular Hydrogen Bonds

**DOI:** 10.3390/molecules22040552

**Published:** 2017-03-29

**Authors:** Poul Erik Hansen, Jens Spanget-Larsen

**Affiliations:** Department of Science and Environment, Roskilde University, Universitetsvej 1, P.O. Box 260, DK-4000 Roskilde, Denmark

**Keywords:** hydrogen bond strength, OH stretching wavenumbers, OH chemical shifts, isotope effects, tunneling splitting, O···O distances in O–H···O systems, Wiberg bond indices

## Abstract

For the purpose of this review, strong hydrogen bonds have been defined on the basis of experimental data, such as OH stretching wavenumbers, ν_OH_, and OH chemical shifts, δ_OH_ (in the latter case, after correction for ring current effects). Limits for O–H···Y systems are taken as 2800 > ν_OH_ > 1800 cm^−1^, and 19 ppm > δ_OH_ > 15 ppm. Recent results as well as an account of theoretical advances are presented for a series of important classes of compounds such as β-diketone enols, β-thioxoketone enols, Mannich bases, proton sponges, quinoline N-oxides and diacid anions. The O···O distance has long been used as a parameter for hydrogen bond strength in O–H···O systems. On a broad scale, a correlation between OH stretching wavenumbers and O···O distances is observed, as demonstrated experimentally as well as theoretically, but for substituted β-diketone enols this correlation is relatively weak.

## 1. Introduction

The nature of the hydrogen bond is an issue of great current interest [1,2,3,4,5,6,7,8,9,10,11]. The title of this paper includes the term “strong intramolecular hydrogen bonds”. Keeping this term is mainly in lack of a better descriptor, but also to refer to already published materials. In this review, “strong” is not equivalent to “low barrier” [3]; in defining a strong hydrogen bond, we follow the recommendations by the International Union of Pure and Applied Chemistry (IUPAC) [4]:

(i) The length of the X–H bond usually increases on hydrogen bond formation leading to a red shift of the infrared X–H stretching frequency and an increase in the infrared absorption cross-section for the X–H stretching vibration. The greater the lengthening of the X–H bond the stronger is the H···X bond”.
(ii) The X–H···Y–Z hydrogen bond … typically include[s] pronounced proton deshielding for H in X–H”.

The definition of a strong intramolecular hydrogen bond can therefore be based on the two abovementioned criteria. However, in the case of XH chemical shifts, these should be corrected for possible ring current effects and anisotropy contributions (see later) [11,12]. Taking this into account, we have adopted an experimental approach for XH = OH: 2800 cm^−1^ > ν_OH_ > 1800 cm^−1^ and 19 ppm > δ_OH_ > 15 ppm (see below). These limits are chosen primarily for practical reasons and may be debated; see for example Exarchou et al. [13]. For selected cases, the present definition is consistent with the p*K*_a_ slide rule definition [2].

Other criteria could be a large ^2^∆C(XD) (two-bond deuterium isotope effects on ^13^C chemical shifts) or ^4^∆C(XD) (four-bond deuterium isotope effects on ^13^C chemical shifts) or large XH primary isotope effects [14]. Isotope effects have the advantage that they are independent of solvent effects and that ring current effects are eliminated. In case of X being nitrogen, a parameter could also be one-bond NH coupling constants, as these decrease with increasing NH bond length. Some of the parameters mentioned above seem to be related. Examples are XH chemical shifts and primary isotope effects [15,16,17], OH chemical shifts, and the OH stretching wavenumber ν_OH_ [18], and ν_OH_ and two-bond isotope effects [19]. This is frequently an advantage: If one parameter cannot be measured, another can be used in describing the hydrogen bonded system.

Measurement of the XH stretching frequencies should be the preferred tool investigating hydrogen bonds. However, as will be discussed, the OH stretching bands may be very broad and the “band center” may be difficult to localize, particularly for strong hydrogen bonds (see, e.g., Figure 1). OH groups may also display broad ^1^H-NMR signals in protic solvents or in aprotic solvents with traces of water [20]. The situation is frequently complicated by the circumstance that many compounds with intramolecular hydrogen bonds are tautomeric. For an asymmetrical system, two bands may be observed causing possible overlap. Tautomeric cases will of course also be covered.

In the present review, we will consider significant developments in recent years as well as central cases from the past. The review will not deal with very low barrier hydrogen bonds, meaning hydrogen bonds giving rise to XH stretching frequencies in the fingerprint region or lower, as these have been reviewed recently by Perrin [3].

Intramolecular hydrogen bonds can be divided into two groups: (i) the donor and acceptor are part of a conjugated system, linking X and Y as in Scheme 1a (also called Resonance Assisted Hydrogen Bonding (RAHB) [21]); and (ii) the donor and the acceptor are not part of such a system, e.g., Scheme 1b (the example shown may also be described as a case of Charge Assisted Hydrogen Bonding (CAHB) [21]). Other situations are seen for example in proteins in which the donor and the acceptor are at different parts of the protein but in close enough proximity to form hydrogen bonds. However, such hydrogen bonds are normally not strong.

According to the definition [4], the greater the lengthening of the X–H bond distance in the X–H···Y system, the stronger and shorter is the H···Y hydrogen bond. Factors important for a short H···Y distance are the bond distance between carbons **1** and **2**, the *a* and *b* angles, and of course the X–H bond length, see Scheme 2. The H···Y distance will be influenced by the three abovementioned factors. The X···Y distance has been used extensively as a hydrogen strength parameter since it is relatively easy to measure in contrast to the X–H and H···Y distances; correlations which are based solely on crystallographic X–H or H···Y distances are, very probably, invalid [22,23,24]. On the other hand, the use of the X···Y distance has been criticized by Perrin [3]. Factors decreasing the *a* and *b* angles are steric strain from substituents at positions 3 or 6. In the latter case, twisting of the RY substituent out of the ring plane may occur [25,26]. Examples of strong steric compression are seen in 1,3,5-triacetyl-2,4,6-trihydroxybenzene (**1**) [27] and in similar systems [26]. Twisting of the RY substituent out of the ring plane is seen in, e.g., 1-acetyl-2-hydroxynaphthalene (**2**) [25]. The importance of directionality has been investigated by Majerz [28].

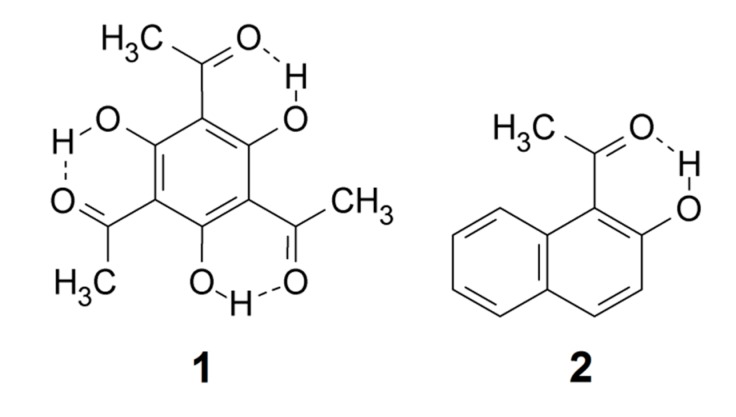



*XH chemical shifts*: It is well known that ring currents may contribute to the chemical shift of hydrogens in the neighborhood of aromatic rings, and therefore also to the chemical shifts of XH taking part in intramolecular hydrogen bonds. For benzene rings this can be estimated from the so-called Johnson-Bovey map in the general case [29]. For large aromatic systems the effects may be larger [30]. Recently, ring current effects have been estimated on OH chemical shifts in a series of intramolecular hydrogen bonded cases [11,12]. Furthermore, for intramolecular hydrogen bonds as described in Scheme 2 an anisotropy effect may also be at play. It was shown that this varies dramatically from C=O to C=S bonds [12]. However, the XH chemical shift corrected for ring current effects and anisotropy effects is a very suitable measure of hydrogen bond strength [12].

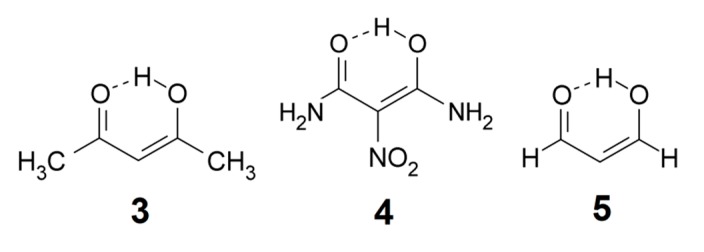



The ^1^H chemical shift of acetyl acetone enol (**3**) is experimentally determined as 15.5 ppm and the anisotropy effect estimated as 0.4 ppm [12] Here, we use the value 15 ppm as a lower limit for strong O–H···Y hydrogen bonds. The upper value 19 ppm is taken from nitromalonamide enol (**4**) [31]. Larger shifts are considered to correspond to systems with very strong hydrogen bonds. OH chemical shifts have in general been calculated in intra-molecular systems with great success [10,27,32] (see Section 2.2).

*XH stretching frequencies.* The upper limit for OH stretching wavenumber in a strong intramolecular hydrogen bond is here taken as 2800 cm^−1^, close to the values observed for malondialdehyde enol (**5**) [33] and acetylacetone enol (**3**) [34]. The lower limit is taken as 1800 cm^−1^; wavenumbers lower than this limit are considered as characterizing very strong hydrogen bonds which are not explicitly considered in this survey.

## 2. Theoretical Predictions

### 2.1. IR Spectroscopy

In recent decades, great progress has been made in the computational prediction of the vibrational structure of molecules. Today, vibrational wavenumbers are routinely predicted to a high degree of accuracy by quantum chemical calculations in the harmonic approximation, accounting for errors due to neglect of anharmonic effects and other shortcomings by the application of semiempirical scaling procedures [35,36,37,38,39,40,41,42,43]. In particular, density functional theory has been found to be useful, with the B3LYP functional [44,45] being the most popular. However, the standard procedures tend to be misleading for strongly hydrogen bonded systems, where an explicit incorporation of anharmonic and dynamical effects in the theoretical model seems to be required. However, accurate treatments are demanding in terms of theoretical knowhow and computational effort [46]. In the following brief account, we focus primarily on examples of the application of approaches beyond the standard harmonic approximation.

As the smallest molecule with an intramolecular OH···O hydrogen bond, malondialdehyde enol ((*Z*)-3-hydroxypropenal, **5**) has been the subject of several advanced theoretical investigations. The IR spectrum displays an OH stretching band with a maximum at 2856 cm^−1^ in the gas phase [33,47] and 2877 cm^−1^ in Ar matrix at 10 K [48]. The hydrogen bonded proton is assumed to be situated in an anharmonic potential corresponding to a symmetrical double minimum potential well; the observed tunneling splitting in the vibrational ground state is 21.58 cm^−1^ [49,50,51]. Theoreticians have mainly focused on calculation of the anharmonic effects on the IR spectrum and on prediction of the tunneling splitting, considering **5** as a convenient test case.

Alparone and Millefiori [52] computed the anharmonic IR spectrum of malondialdehyde enol (**5**) by the Vibrational-Self-Consistent-Field (VSCF) and the correlation-corrected VSCF (CC-VSCF) techniques [53,54,55]. They found that the anharmonic contributions were substantial, especially for the OH stretching vibration by reducing the harmonic value by more than 500 cm^−1^. The OH stretching mode was predicted to couple substantially with other modes, such as CH stretching and OH in-plane bending.

Tayyari and coworkers [56] constructed a two-dimensional potential energy function, leading to coupling of OH stretching and OH bending modes. The predicted tunneling splitting at the MP2 and B3LYP levels were in good agreement with the observed splitting. The authors argued that the coupling between OH stretching and in-plane-bending modes explains the anomalous vibrational behavior of bent hydrogen bonded systems.

Using a previously proposed full dimensional (21D) potential for malondialdehyde enol (**5**) [57], Manthe and coworkers [58] applied accurate full-dimensional quantum dynamics methods: the multi-configurational time-dependent Hartree approach, and a diffusion Monte-Carlo based spectral evolution method, both methods yielding satisfactory agreement with the observed data.

Bowman and associates [59,60] computed an accurate full-dimensional ab initio potential energy surface for malondialdehyde enol (**5**), based on high-level electronic energies (CCSD(T)). They obtained a barrier for intramolecular proton transfer equal to 4.1 kcal/mol. By using this potential they performed model one-dimensional calculations, as well as full-dimensional diffusion Monte-Carlo based calculations. The latter predicted a tunneling splitting in excellent agreement with experiment.

Schröder and Meyer [61] investigated the tunneling splitting in low-lying vibrationally excited states of malondialdehyde enol (**5**) by the multi-configuration time-dependent Hartree method, using the full-dimensional potential energy function previously published by Bowman and associates [59]. They obtained good agreement with experimental values for several states. Additional references to recent work in this field may be found in the literature cited.

Application of the abovementioned advanced procedures to systems larger than malondialdehyde enol (**5**) is not straightforward. In general, approximate theoretical methods must be applied. Mavri and Grdadolnik [62,63] investigated the proton transfer dynamics in acetylacetone enol (**3**), the simplest member of the series of β-hydroxyketone enols. They applied a mixed quantum-classical approach, treating the proton involved in the medium-strong intramolecular hydrogen bond as a quantum particle, while the rest of the system was treated using classical mechanics. Molecular dynamics calculations lead to prediction of the general structure of the observed, very broad OH stretching band with a maximum around 2800 cm^−1^ (see Figure 1).

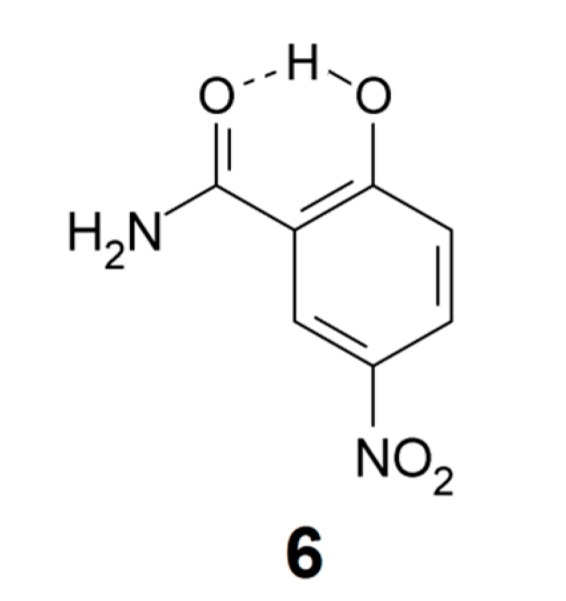



In more recent work, Mavri and coworkers have investigated biologically active compounds. For example, they applied Car-Parrinello [64] molecular dynamics calculations in a simulation of the vibrational spectrum of 2-hydroxy-5-nitrobenzamide (**6**) in the crystal phase, a system with strong intra- and intermolecular hydrogen bonding [65]. In an analysis of the importance of vibrationally enhanced catalysis, Kamerlin, Mavri, and Warshel [66] concluded that dynamical effects and tunneling are irrelevant for enzyme catalysis. Car-Parrinello molecular dynamics calculations were also applied by Durlak et al. [67,68,69] in investigations of the vibrational structure of compounds like nitromalonamide enol (**4**) and 2-acetyl-1,8-dihydroxy-3,6-dimethylnaphthalene (**7**), and by Panek et al. [70] in a study of intramolecular hydrogen bonds in a series of 8-hydroxyquinoline *N*-oxide (**8**) derivatives.

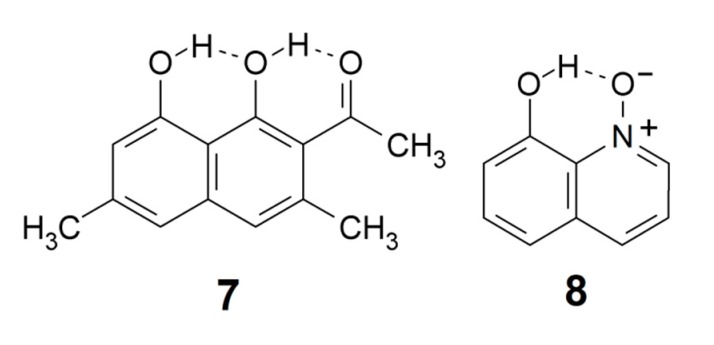



Došlić and coworkers considered the vibrational spectrum of acetylacetone enol (**3**) in considerable detail by using reduced-dimensional quantum chemical models [71,72] and second-order perturbation theoretical calculation of anharmonic effects on the vibrational wavenumbers [73] (PT2, see below). The authors concluded that the considerable broadening of the observed OH stretching band is largely a consequence of the torsional dynamics of the methyl groups.

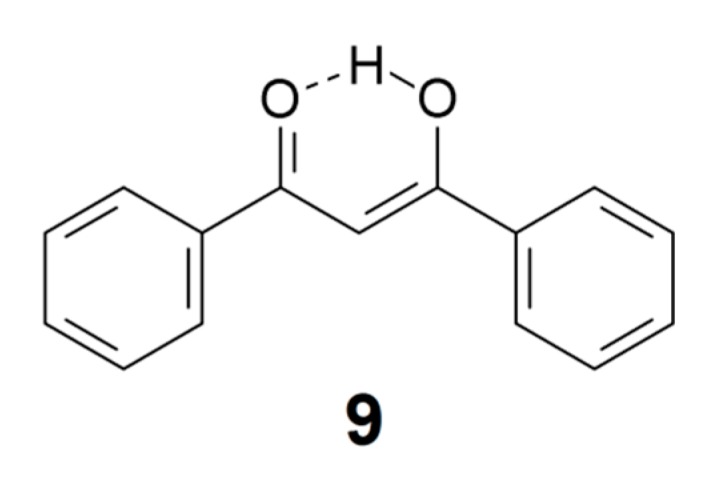



Replacing the methyl groups in acetylacetone enol with phenyl groups leads to dibenzoylmethane enol (**9**). The intramolecular hydrogen bond in this molecule is short, with an O···O distance around 2.46 Å [74]. The OH stretching band of **9** is believed to be situated around 2600 cm^−1^ (see below). The IR spectrum of this compound has long puzzled spectroscopists because of the broad and irregularly shaped band between 1700 and 1400 cm^−1^ with huge integrated intensity, characterized by strong Evans (or Fermi) windows [75,76]. This “monster band” is probably associated with the OH bending motion coupled with other vibrational motions in this spectral region [75,76], but a full understanding of the structure of this band still awaits the results of an adequate theoretical analysis.

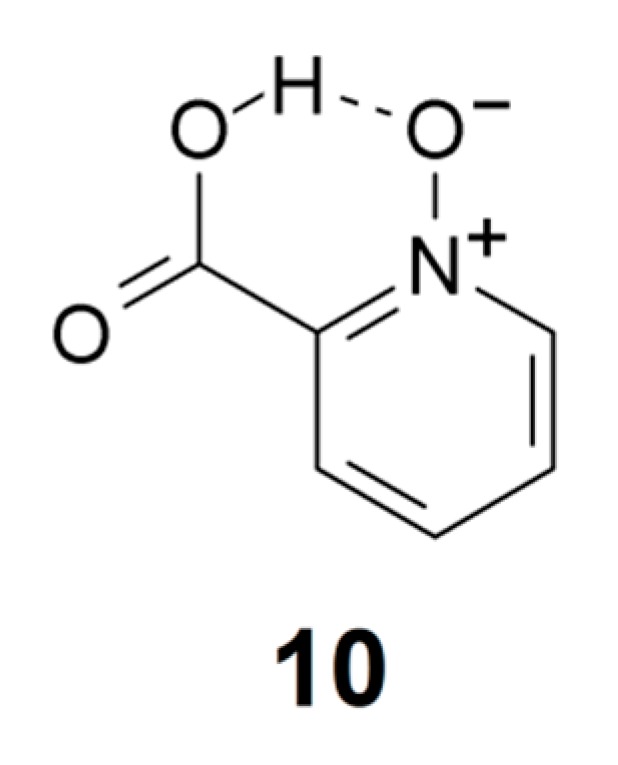



Szczepaniak, Person, and Hadži [77] performed a simplified but remarkably efficient anharmonic analysis of the IR spectrum of picolinic acid *N*-oxide (**10**), yielding a satisfactory interpretation of the entire spectrum. The analysis indicated that the OH stretching coordinate contributes to several normal modes, mixing extensively with other internal coordinates, in particular OH bending and C=O stretching. Unfortunately, general application of this procedure is not straightforward.

Barone and coworkers [78,79] developed a fully automated second-order perturbation theoretical (PT2) approach to the prediction of anharmonic wavenumbers. This procedure is implemented in the Gaussian software package [80] and the application is straightforward. However, the PT2 calculation tends to be extremely time-consuming, application to large molecules is generally impractical. Spanget-Larsen et al. [18] performed PT2 calculations for a large number of hydroxy compounds, including “free” as well as hydrogen bonded groups, and compared the computed wavenumbers with those obtained within the corresponding harmonic approximation. B3LYP density functional theory was applied, and in order to facilitate the PT2 calculations for several quite large molecules, a fairly modest basis set was applied: 6-31G*. It was found that the anharmonic OH stretching wavenumbers were essentially linearly related to those predicted within the harmonic approximation [18]. Moreover, a linear relationship, P(Harm), was established between the observed ν_OH_ wavenumbers, ranging from 3600 to 1900 cm^−1^, and those predicted by a routine B3LYP/6-31G* harmonic analysis [18]:

P(Harm): ν_OH_{obsd} = −757 + 1.171 × ν_OH_{harm} (1)

(32 data points: R = 0.989, SD = 74 cm^−1^). A standard deviation of 74 cm^−1^ seems quite acceptable in view of the experimental uncertainties associated with several of the observed wavenumbers [18]. Wavenumbers predicted by this regression equation will be denoted P(Harm).

The P(Harm) procedure has turned out to be quite useful. For example, Buemi and Zuccarello [81] computed an anharmonic PT2 wavenumber ν_OH_ = 1428 cm^−1^ for the very short intramolecular hydrogen bond in nitromalonamide enol (**4**), but the P(Harm) value is 1920 cm^−1^ [18]. The P(harm) prediction was subsequently supported by the results of sophisticated molecular dynamics calculations [67]. In fact, the PT2 procedure appears to overestimate the wavenumber shifts due to strong intramolecular hydrogen bonding, leading to the prediction of too low OH stretching wavenumbers [18]. Dziembowska et al. [82] investigated the vibrational spectrum of 8-hydroxyquinoline *N*-oxide (**8**) which displays a complex band system between 3000 and 2000 cm^−1^. They assigned a feature at 2330 cm^−1^ to the OH stretching band, but the P(Harm) value is 2877 cm^−1^ [19]. The P(Harm) prediction was confirmed by correlation with NMR parameters [19] and was subsequently supported by the results of an advanced molecular dynamics simulation [70].

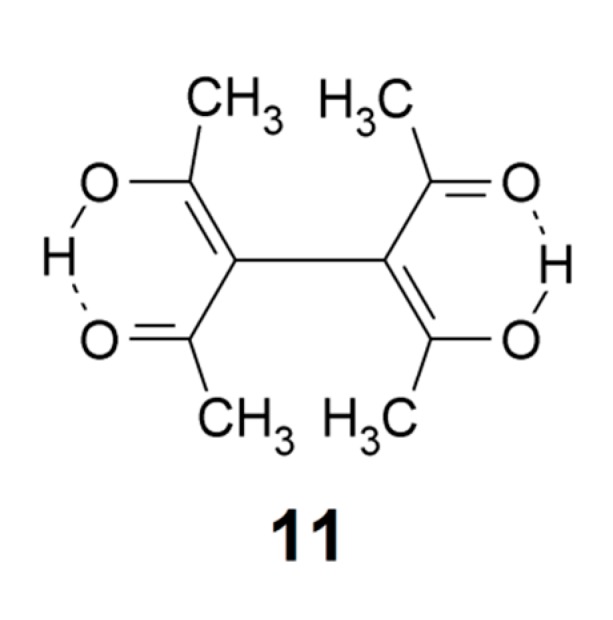



As indicated above, dibenzoylmethane enol (**9**) has a short intramolecular hydrogen bond and its IR spectrum of has been a subject of debate [75,76]. The P(Harm) procedure predicts an ν_OH_ value of 2643 cm^−1^ [18], which is consistent with the assignment by Tayyari et al. [76] of a weak and broad feature around 2600 cm^−1^. The enol tautomer of tetra-acetylethane (**11**) has two even shorter intramolecular hydrogen bonds [83]. Guided by the simple relationship between OH stretching frequency and O···O distance suggested by Bellamy and Owen [84], Emsley et al. [83] assigned an IR feature around 1500 cm^−1^ to the OH-stretches (similar assignments were made for a series of 3-substituted 2,4-pentanediones). However, Raissi et al. [85] more recently assigned a weak and broad IR feature of **11** close to 2630 cm^−1^. The P(harm) values are 2651 and 2660 cm^−1^, consistent with the assignment of Raissi et al. [85]. It would seem that the Bellamy-Owen equation [84] seriously underestimates the OH stretching wavenumbers for this type of intramolecular hydrogen bonding. The relationship between OH stretching frequency and hydrogen bond distance has been explored by a number of authors, primarily for intermolecular hydrogen bonding in crystals and hydrates [84,86,87,88,89,90,91,92]; see Bratos et al. [93] for references to recent literature. However, application of the relationships proposed in these publications to intramolecular hydrogen bonding in organic molecules is probably not straightforward.

The P(Harm) procedure was recently applied by Vojta et al. [94] in an investigation of flavonoids containing several hydroxy groups. They found that the preferred molecular shape of a gluco-flavonoid like myricitrin was determined by cross-linking intramolecular hydrogen bonds. Unfortunately, the broad and intense absorption in the OH stretching region prevented precise IR assignments.

The P(Harm) Equation (1) based on the results of a B3LYP/6-31G* harmonic analysis evidently yields satisfactory predictions of observed OH stretching wavenumbers for a variety of systems, even using a modest basis set. The collected B3LYP/6-31G* data for systems with intramolecular OH···O=C hydrogen bonding [18] thus offer interesting opportunities for the study of possible correlations between internal theoretical parameters. A crude correlation is found between the calculated ν_OH_ wavenumbers and the calculated O···O distances for hydrogen bonded systems (33 data points: R = 0.88, SD = 165 cm^−1^); see also the discussion in Section 6. A better correlation is observed between calculated ν_OH_ wavenumbers and H···O distances for the same set of systems (33 data points: R = 0.96, SD = 93 cm^−1^). Perhaps not surprisingly [91], an excellent correlation is found between calculated ν_OH_ wavenumbers and O–H distances. The full set of data, including also “free” OH groups, yields a slightly curved second order polynomial fit as shown in Figure 2 (39 pts: R = −0.999, SD = 17 cm^−1^). A similar correlation comprising 22 OH groups was observed by Vojta et al. [94]. This means that irrespective of whether the OH group is “free” or engaged in hydrogen bonding, and independent of other structural details, the computed OH stretching wavenumber can be predicted from the computed O–H bond distance to an accuracy of about 20 cm^−1^.

Finally, an excellent linear correlation is observed between calculated ν_OH_ wavenumbers and Wiberg bond indices [95,96] for H···O distances, as shown in Figure 3 (33 data points: R = −0.996, SD = 30 cm^−1^). The Wiberg bond indices were computed in a basis of natural atomic orbitals (NAO) [97] using the Gaussian software package [80]. The Wiberg bond index is designed as a measure of covalent bonding. The excellent correlation in Figure 3 indicates a tendency towards increased covalent character when moving from weaker to stronger hydrogen bonds. The question of the covalency of hydrogen bonding has recently been discussed by several authors [7,8,10].

### 2.2. NMR Spectroscopy

DFT calculations of NMR nuclear shielding (chemical shifts) have reached a high degree of accuracy. The use is already discussed for calculation of iso-chemical shifts in the estimation of ring current effects in relation to OH chemical shifts and their use in determination of hydrogen bond strength [12]. One is also able to calculate changes in nuclear shieldings, which is the first factor in the calculation of isotope chemical shifts. Such calculations will normally predict the signs correctly, whereas the magnitudes will depend on estimating the correct change in the bond length upon deuteriation; for an example, see Grech et al. [98]. For applications of DFT theory in calculations of ^1^H chemical shifts of intramolecular hydrogen bonds, see the work by Siskos et al. [10,32]; these authors use OH chemical shifts to predict the OH proton positions [10]. Most recently, Scheiner [11] has published a computational analysis of the downfield shift of the NMR signal of the bridging proton in a hydrogen bond. There is an interplay between the shielding effects caused by charge transfer and polarization due to formation of the hydrogen bond, and those due to the mere presence of the proton-accepting group. According to Scheiner [11], this second positional shielding must be subtracted from the full observed shift in order to evaluate the deshielding of the proton caused purely by hydrogen bond formation.

## 3. β-Hydroxy Carbonyl Compounds

This class of compounds contains numerous species with strong intramolecular OH···O=C hydrogen bonding and they have been investigated extensively. Early developments were reviewed comprehensively in the classical account by Emsley [99], and a recent treatise was published by Gilli and Gilli [1]. Examples involving phenolic hydroxy groups were reviewed by Hansen and Spanget-Larsen [100]. OH stretching wavenumbers, proton chemical shifts (δ_H_), and two-bond deuterium isotope effects (^2^∆C_OD_) for a large series of a variety of β-hydroxy carbonyl compounds were collected and analyzed by Spanget-Larsen et al. [18] and by Hansen and Spanget-Larsen [19].

Among recent experimental advances must be emphasized the results of Suhm and associates [101,102]. These workers applied a combination of FTIR and Raman spectroscopy in supersonic jets and in rare gas matrices in an investigation of the vibrational structure of malondialdehyde enol (**5**). They found that the hydrogen transfer through the substantial barrier between the two equivalent enol tautomers is accelerated by more than a factor three by OH bending excitation in phase with suitable motions of the molecular backbone, thereby emphasizing a strong coupling between hydrogen transfer and vibrational excitation. The authors established a very large tunneling splitting of 69 cm^−1^ for the OH bending fundamental. The experimental results were rationalized by comparison with the results of B3LYP and MP2 calculations.

## 4. β-Hydroxy Thiocarbonyl Compounds

Thioketones tend to be rather unstable but a number of β-hydroxy thioketones are stabilized by strong intramolecular OH···S=C hydrogen bonding. Particular interest has been devoted to the molecular and vibrational structure of monothioacetylacetone enol (**12**) and its derivatives [103,104,105,106]. The OH stretching bands in these compounds are generally weak and diffuse and they frequently escape detection. OH stretching wavenumbers have been reported for monothioacetylacetone enol (**12**) [104] and *p*-methyl(thiobenzoyl)acetone enol (**13**) [105], 2490 and 2571 cm^−1^, respectively. As the β-thioxoketones are tautomeric, the NMR chemical shifts are average values. Even an open form may be involved which further complicates matters. However, using low temperature measurements both ^1^H- and ^13^C-NMR data for the thione form have been estimated; the OH chemical shifts for **12**, **13** and **14** are 15.2, 16.4 and 17 ppm, respectively [107].

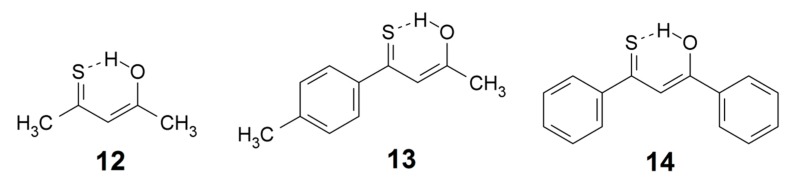



Great effort has been devoted in order to understand the observed photochromic properties associated with the OH···S=C system of monothioacetylacetone enol (**12**) and its derivatives. The structure of the photoproduct was long a subject of debate, but the question was solved by IR spectroscopy of the product trapped in a cryogenic matrix in combination with theoretical calculations: the isolated photoproduct was identified as the *exo*-SH rotamer of the enthiol tautomer [103]. The individual steps of the photochromic transformation of monothiodibenzoylmethane enol (**14**) were recently investigated by combining laser photolysis with NMR detection: Direct laser irradiation of a sample located in the NMR magnet allowed in situ monitoring of phototransformation products [108].

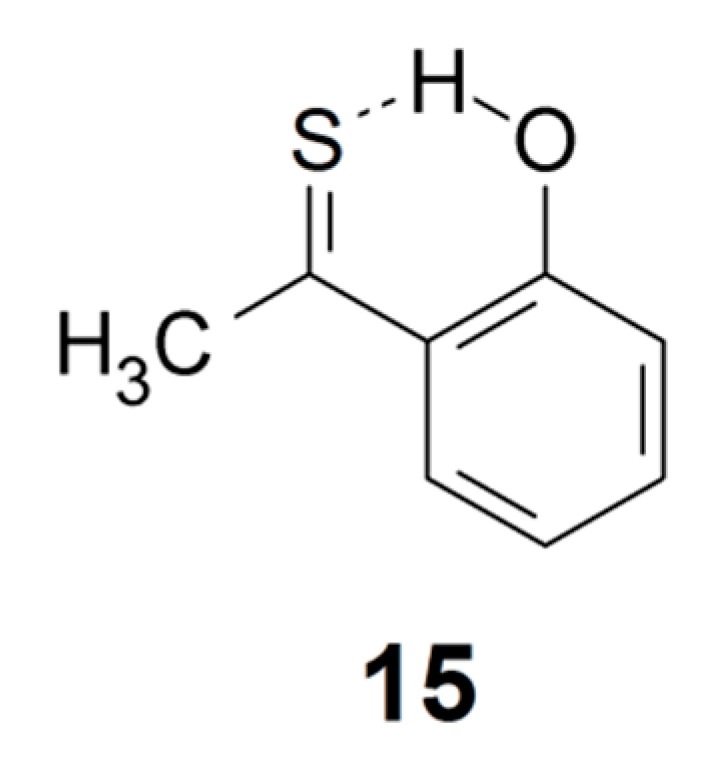



Duus and coworkers [109] have described the preparation of a new class of stable thiocarbonyl compounds: *ortho*-hydroxythioacetophenones. The OH stretching wavenumber and δ_H_ value for 2-hydroxythioacetophenone (**15**) were reported as 2900 cm^−1^ and 13.35 ppm, indicating medium strong intramolecular hydrogen bonding [18,109].

## 5. Intramolecular Hydrogen Bonds without a Double Bond Linker between the Donor and the Acceptor

The protonated 1,8-bis(dimethylamino)naphthalenes (DMANs, see Scheme 1b) show strong hydrogen bonds, judging from the ^1^H chemical shifts which are found in the range from 17.28 to 20.6 ppm [98,110]. One-bond deuterium isotope effects on ^15^N chemical shifts are small in the symmetric cases [98], again underlining that one is dealing with a strong hydrogen bond. Primary deuterium isotope effects [98,110] are seen to vary from 0.7 ppm to 0.14 ppm. The stronger is the hydrogen bond, the smaller is the primary isotope effect. Chmielewski et al. [110] have plotted primary deuterium isotope effects for both symmetric and asymmetric compounds vs. the NH chemical shifts. However, this creates a problem as values for unsymmetrical compounds contain an equilibrium contribution [14]. If this contribution were taken into account, an even better fit would probably be found.

A comparison of data from plots 4 and 5 in the publication by Hansen et al. [111] clearly shows that the coupling constant ^1^J(N,H) is decreasing as the hydrogen bonding is becoming stronger. A change in NH chemical shift of ~2 ppm leads to a change in the coupling constant of ~2 Hz. For ammonium ions, a coupling of –74 Hz is found. A similar value is seen in the anilinium ion. For DMANs, ^1^J(N,H) has dropped to ~60 Hz [98,112] which is in accordance with the change in NH chemical shift.

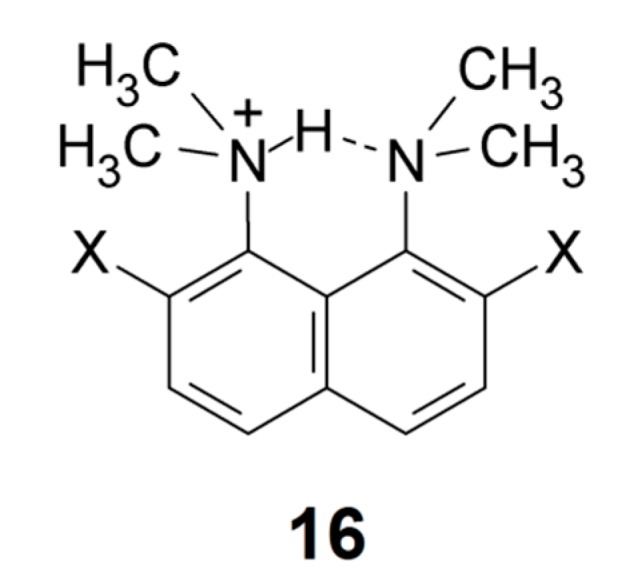



From IR spectra the NH frequency was determined for two protonated DMANs, the 2,7-dichloro and the 2,7-dibromo derivative (**16**) with Br^−^ as the counter ion. For the dibromo derivative the NH stretching wavenumber was determined as 560 cm^−1^ [113], whereas for the dichloro derivative it was found at 494 cm^−1^ (center of gravity at 530 cm^−1^). One would possibly expect the former to have the lowest frequency, as the NH chemical shift is lower for the dichloro derivative (20.13 vs. 20.27 ppm). These compounds clearly fall outside the present range of strong hydrogen bonds, which is evidenced both from the NMR and IR data. Bartoszak et al. [114] published the spectrum of DMANH^+^, ClO_4_^−^ without further analysis. The very strong band observed in the two derivatives above is not seen here. However, a broad band is found around 2000 cm^−1^.

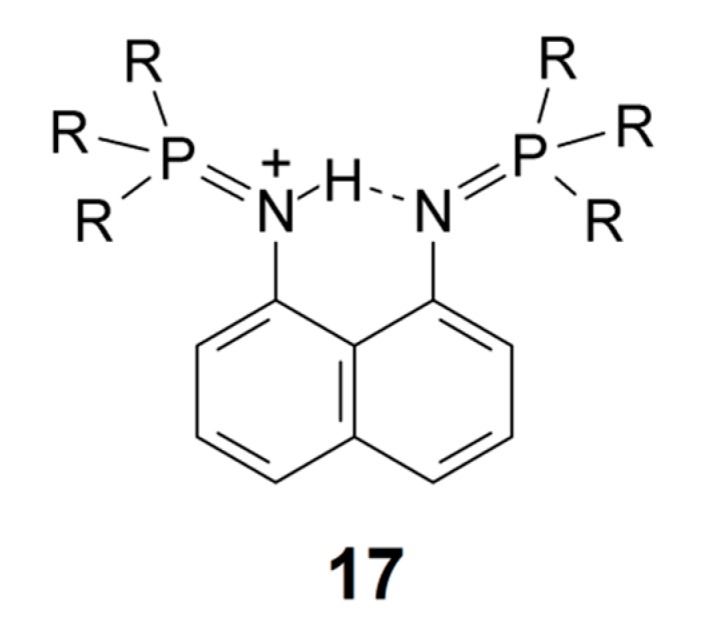



Bisphosphazene proton sponges (**17**) show NH chemical shifts in the range from 13.3 to 15.8 ppm. The lower values are due to R = isotropyl or R = cyclopentyl, and the higher values due to R = methyl and R = butyl. In the former cases, the N···N distances range from 2.63 to 2.64 Å, whereas, in the latter cases, the range is from 2.57 to 2.59 Å [115].

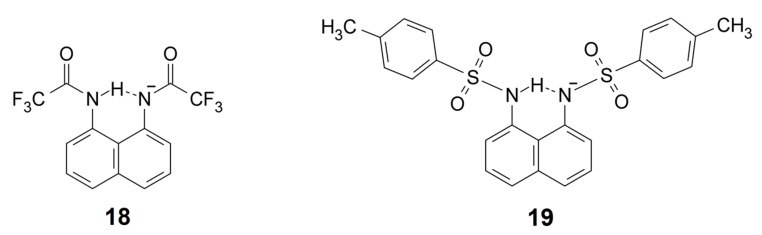



Similar systems are represented by *N*,*N*-(naphthalene-1,8-diyl)bis(2,2,2-trifluoroacetamide) (**18**) and 1,8-bis(4-toluensulphonamido)naphthalene (**19**), except they involve a negatively charged amido nitrogen. A DMANH^+^ cation is the counter ion in this case. The ^1^H chemical shifts of the NH protons are 15.6 and 15.1 ppm for **18** and **19** [116].

Another group of compounds without resonance assistance are dicarboxylic acid anions, Scheme 3. This type of compounds has been reviewed by Perrin and Nielson [117]. Sigala et al. [118] found for salicylate anions (**20**) substituted at the benzene ring chemical shifts from 15 to 18 ppm. A rather clear correlation was found between OH chemical shifts and primary isotope effects, p∆H(D). OH chemical shifts were also correlated to ∆p*K*_a_ and this parameter was linearly correlated to O···O distances and ∆G_f_ (hydrogen bond formation energy).

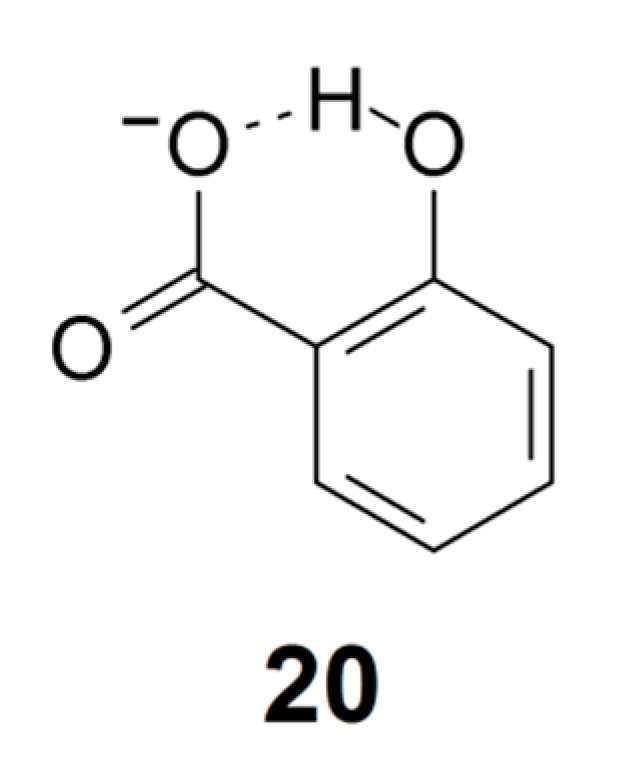



In *ortho*-Mannich bases, a strong intramolecular hydrogen bond is formed between a phenolic OH group and an *N*,*N*-dialkylaminomethyl substituent in the *ortho*-position (**21**). They have been studied intensely by both IR and NMR techniques for many years [100,119,120]. These compounds are excellent models for the investigation of intramolecular proton transfer phenomena. Proton transfer leads to a phenolate tautomer with formally separated charges (**22**) (a similar situation may apply for Schiff bases [100]). Charge separation is clearly important in understanding the factors influencing proton transfer and the way the equilibrium responds to solvent and temperature.

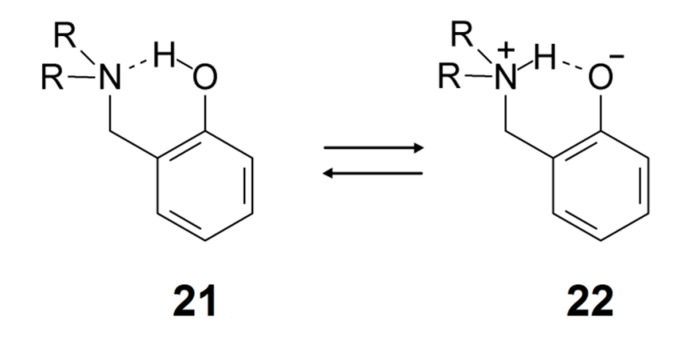



Recently, Mannich bases of 3,4,5,6-tetrachlorophenol and related species were investigated in considerable detail [121]. Optical and NMR spectroscopic studies showed that lowering of the temperature favors the proton-transfer form **22** [121,122]. Intrinsic deuterium isotope effects on ^13^C chemical shifts in the phenol-form **21** were estimated based on OH bond lengths, and the observed deuterium isotope effects on ^13^C chemical shifts were demonstrated to be largely of equilibrium type except at ambient temperatures [121]. The IR spectra of *ortho*-Mannich bases are frequently complicated, giving rise to broad and intense structures in the OH stretching region, but a convincing correlation between wavenumbers of observed band centers and B3LYP/6-31G* harmonic wavenumbers were established for a series of compounds: ν_OH_{obsd} = −1290.2 + 1.2783 × ν_OH_{harm} [121]. The correlation equation is thus different from the one observed for OH···O=C systems, i.e., Equation (1) [18,19].

## 6. Summary and Perspectives

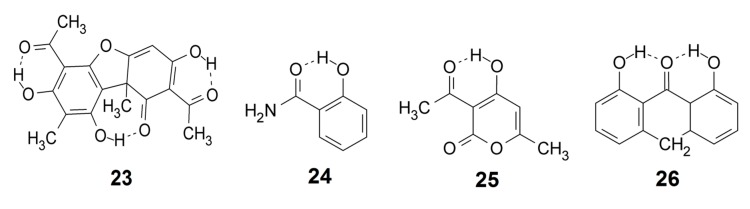



The present review has defined strong hydrogen bonds from an experimental angle based on OH stretching frequencies 2800 > ν_OH_ > 1800 cm^−1^ and OH chemical shifts 19 ppm > δ_OH_ > 15 ppm. Some authors have intentionally avoided use of the term “hydrogen bond strength” [118], but without setting something else instead. One parameter often mentioned as a measure of hydrogen bond strength is the heavy atom distance X···Y. This has been criticized by Perrin [3].

A plot of observed OH stretching frequencies vs. the corresponding observed O···O distances is seen in Figure 4 for a number of compounds, such as **1**, **3**, **4**, **8**, usnic acid (**23**), salicylamide (**24**), dehydracetic acid (**25**), anthralin (**26**), etc., as well as data from Bertolasi et al. [123]. The general trend is a fair correlation between ν_OH_ distance and O···O distance, but clearly with some large individual variations. The point falling far below the correlation line is due to nitromalonamide enol (**4**), and the one above the line is due to salicylamide (**24**). The data points above 2900 cm^−1^ do not correspond to strong hydrogen bonds but are added to give a full picture. The data from Bertolasi et al. [123] refer to substituted dibenzoylmethanes; a very weak dependence of the OH stretching wavenumber on the O···O distance is seen, giving rise to a horizontal row of data points, indicated by black dots in Figure 4. Conjugation changes the O···O distance but apparently not the OH stretching wavenumber. The data point just above this row of data is due to acetylacetone (**3**) and above and below the row are data from usnic acid (**23**) which in the C-ring is a conjugated acetylacetone but with steric interactions. The neighboring point is from **1**, which is also strongly sterically hindered.

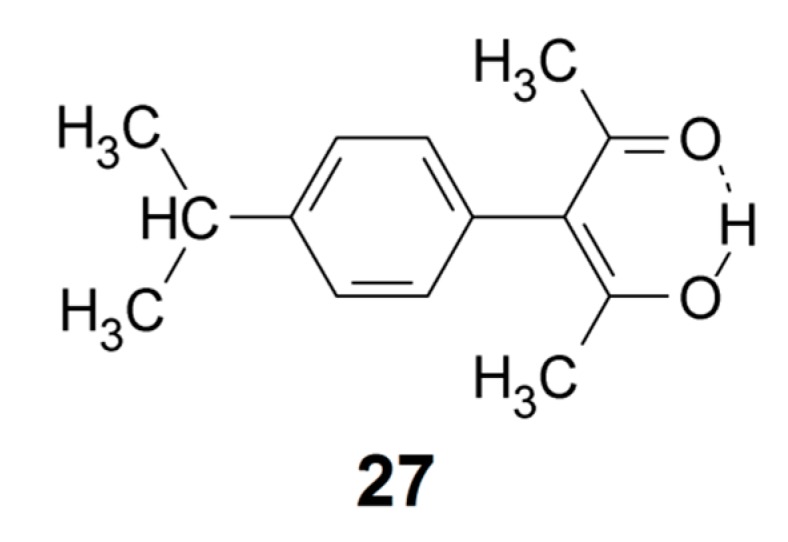



The number of data points is presently too small to make a very detailed analysis. More data points can possibly be obtained by scrutinizing data in the literature that have not been assigned using Equation (1). Such an example is 3-(4-isopropylphenyl)acetylacetone (**27**) with an O···O distance of 2.419 Å [83]. An IR absorption band around 1500 cm^−1^ was assigned by Emsley et al. to the OH stretching [83], but the P(Harm) prediction is 2682 cm^−1^, at variance with this assignment. However, weak and broad absorptions were observed at 2573 and 2329 cm^−1^ [83], and it seems likely that the OH stretching band is situated in this region of the spectrum, similar to the case of tetra-acetylethane enol (**11**) [85]. This assumption is supported by the correlation in Figure 4.

Sidorkin et al. [124] have suggested a bifurcated hydrogen bond situation for acetylacetone enol interacting with Lewis bases. Mori and Masuda [125] have investigated the influence of solvents on the intramolecular hydrogen bond in dibenzoylmethane enol (**9**). Using a similar approach as Sidorkin et al. [124], Mori and Masuda found a slight solvent induced change of the O–H bond length. For acetylacetone enol (**3**) the OH chemical shift in CDCl_3_ is 15.46 ppm; it is almost unchanged in DMSO-*d*_6_, 15.58 ppm, and even in DMF-*d*_7_ the change is very small, 15.78 ppm. An idea could be to use an external perturbation to define the strength of the intramolecular hydrogen bond. Apparently, the intramolecular hydrogen bond in acetylacetone enol (**3**) is not broken by interaction with hydrogen bonding solvents like DMSO and DMF, consistent with the definition of a strong intramolecular bond in this compound. However, the OH chemical shift may not be a sensitive indicator as the OH may still be taking part in a strong intermolecular hydrogen bond. Similar questions have been addressed by Gerothanassis and coworkers [13,32] in investigations of flavonoids with intramolecular hydrogen bonds. The CH_3_ chemical shift in acetyl acetone enol (**3**) could possibly be a better measure, because the breaking of the O–H···O=C hydrogen bond, due to formation of an intermolecular hydrogen bond with the solvent, would most likely lead to a rotation of the acetyl group. However, the CH_3_ chemical shift is unperturbed. For 2-hydroxy-4-methoxyacetophenone (**28**) the OH chemical shift is 12.75 ppm. The OH chemical shift changes only slightly in going from CDCl_3_ to DMF-*d*_7_. On the other hand, the H-6 chemical shift does. This might indicate a rotation of the acetyl group and a breaking of the hydrogen bond in DMF. To use this concept more experiments need to be carried out.

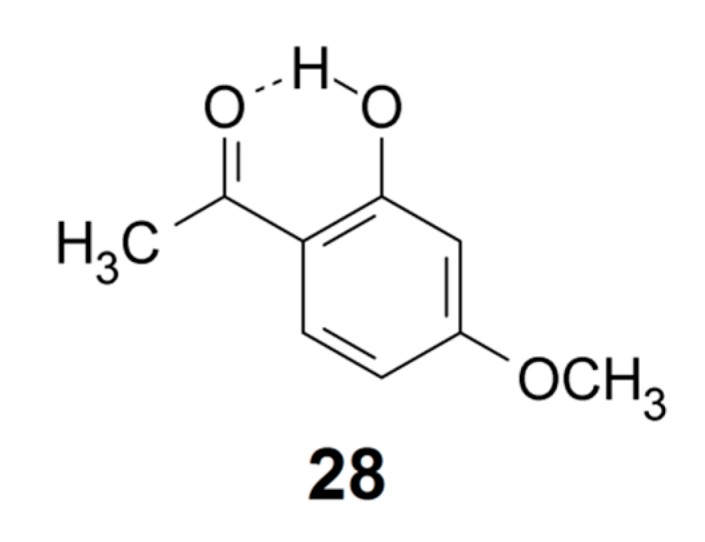



## Figures and Tables

**Scheme 1:**
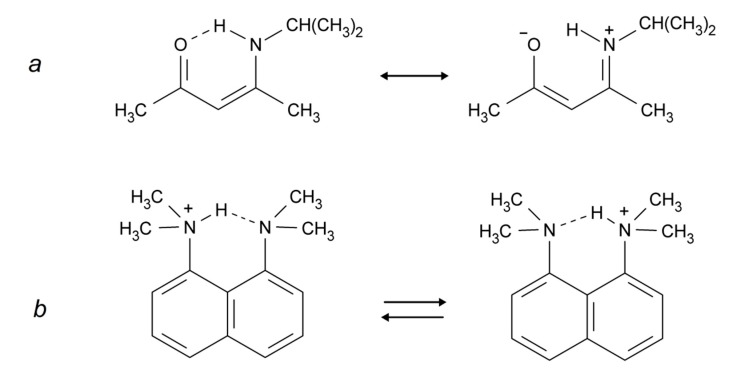
(**a**) RAHB (Resonance Assisted Hydrogen Bonding) system; (**b**) non-RAHB system.

**Scheme 2:**
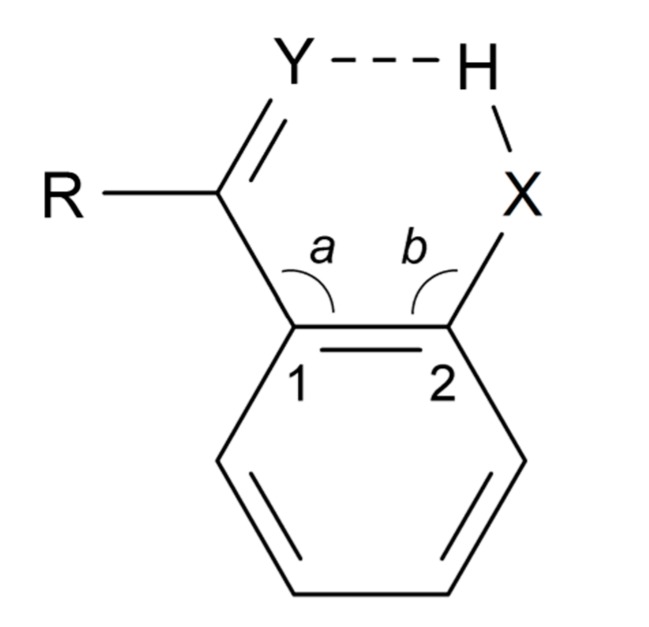
Geometrical parameters in a typical benzene derivative with intramolecular hydrogen bonding.

**Figure 1:**
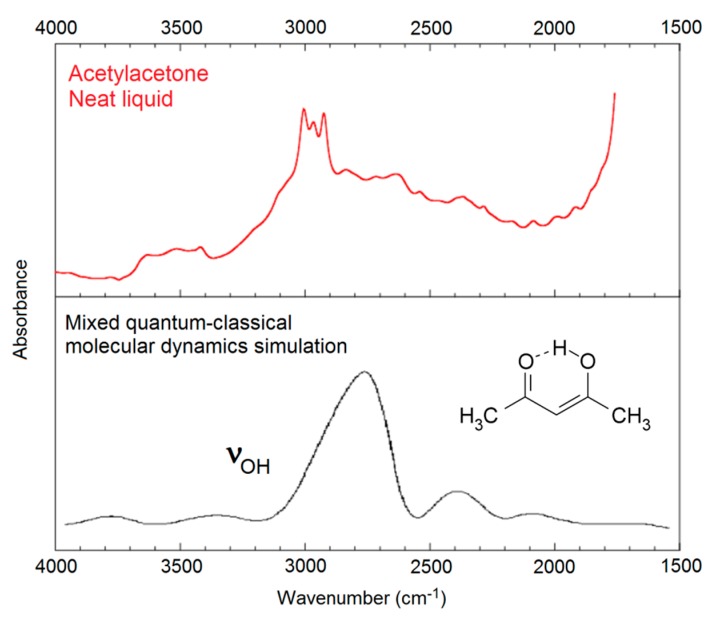
The OH stretching region of the IR spectrum of acetylacetone. Top: Observed spectrum. Bottom: Simulated OH stretching band of the intramolecularly hydrogen-bonded enol form (**3**) of acetylacetone, adapted with permission from Mavri and Grdadolnik [63] (copyright © 2001, American Chemical Society). The observed spectrum contains contributions from the diketoform of acetylacetone, and from CH-stretching bands and summation bands, contributions which are not included in the theoretical simulation.

**Figure 2:**
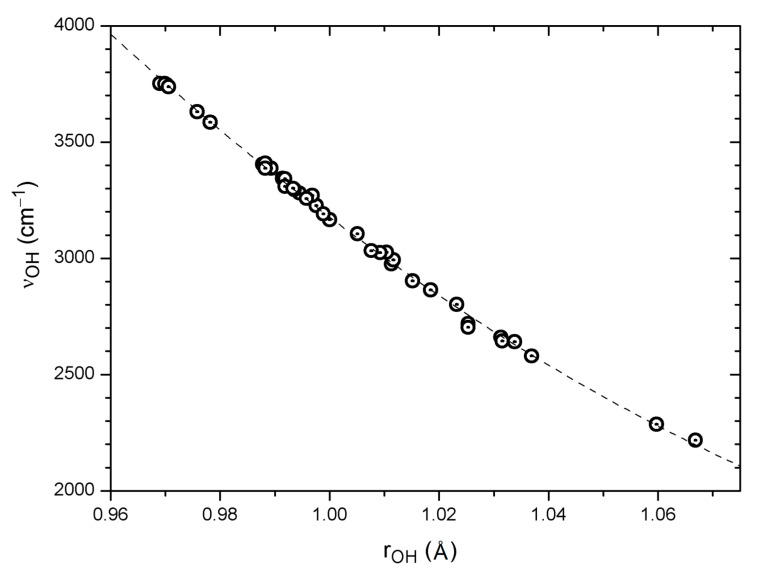
Correlation of calculated harmonic ν_OH_ wavenumbers with the corresponding OH distances for 39 OH···O=C systems, including also a few non-hydrogen bonded OH groups (see text). The regression curve is *Y* = 68,030.6 − 111908.717 · *X* + 47,055.135 · *X*^2^ (39 pts: R = –0.999, SD = 17 cm^−1^).

**Figure 3:**
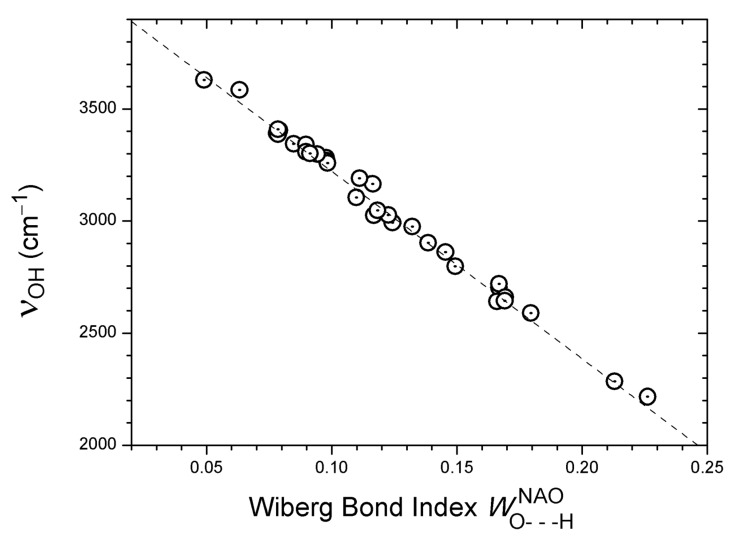
Correlation of calculated harmonic ν_OH_ wavenumbers with computed Wiberg bond indices for the corresponding H···O distances, considering 33 OH···O=C systems (see text). The regression line is *Y* = 4057.6 − 8257.546 · *X* (33 pts: R = −0.996, SD = 30 cm^−1^).

**Scheme 3:**
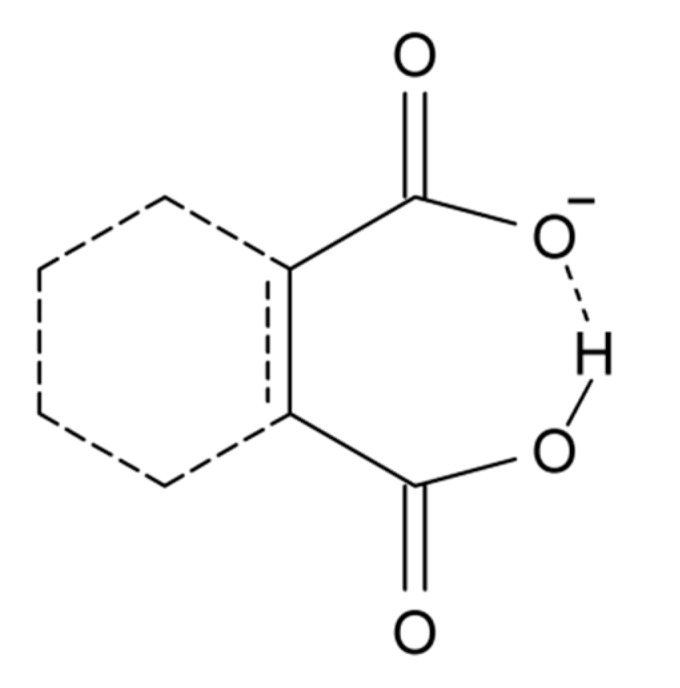
The diagram covers maleic acid anion, phthalic acid anion, etc.

**Figure 4:**
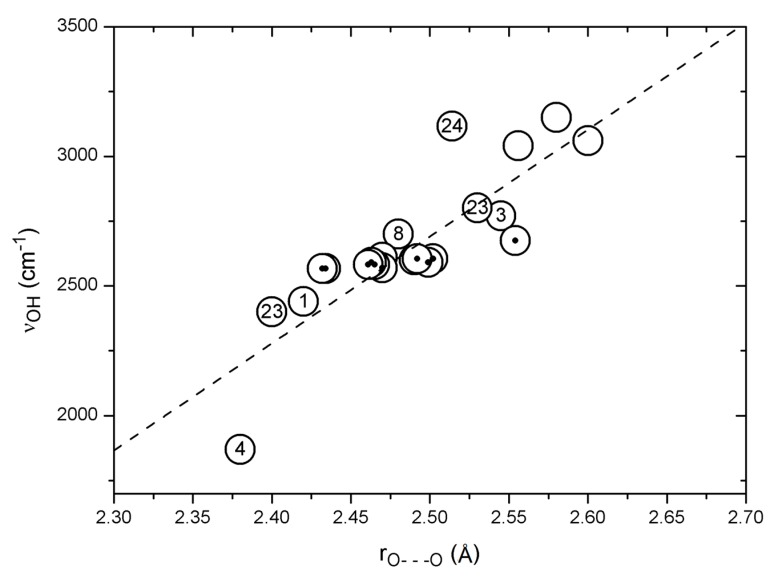
Correlation between observed OH stretching wavenumbers and observed O···O distances (see text). Points from Bertolasi et al. [123] are marked with dots. The correlation line is *Y* = −7616 + 4122.9 · *X* (22 pts: R = 0.83, SD = 161 cm^−1^).
